# Alarm performance in a novel continuous glucose monitor

**DOI:** 10.1186/cc9828

**Published:** 2011-03-11

**Authors:** R Gottlieb, N Yang, F Luo, C Chiu, R Joshi, S Smith

**Affiliations:** 1 Medtronic, Northridge, CA, USA

## Introduction

The ability of a continuous glucose monitor (CGM) to provide actionable information is of utmost importance in the critical care setting. Medtronic has developed a new CGM specifically for the hospital environment. A human feasibility trial was conducted to assess the performance of this system in the ICU. While often overlooked, alarm performance is essential to characterizing both the safety and value of any clinical system - especially in a critical care setting. An analysis was conducted on the sensitivity and specificity of the alarm algorithm to simulate the expected performance when used in the clinical setting.

## Methods

A feasibility study was targeted to enroll 10 ICU patients for 72 hours in the surgical ICU of an academic institution. Enrollment was determined by two consecutive glucose values greater than 140 mg/dl. The sensor data were collected while blinded to the clinicians and hourly reference glucose data were collected using a chemistry analyzer (YSI 2300D). Data analysis was performed to mimic a clinical setting that samples glucose every 6 hours. Sensors were calibrated with four reference values per day. Paired sensor glucose values and YSI values were analyzed for sensitivity and specificity of alarm setting (100 to 140 mg/dl) based on a target range of 100 to 140 mg/dl. For sensitivity and specificity, hypoglycemia was defined as 90 mg/dl or less, and hyperglycemia was defined as 160 mg/dl or greater. This analysis was repeated for paired sensor glucose values and point-of-care meter values in the same alarm settings and target range.

## Results

Two patients completed 72 hours at the time of this abstract. There were not enough paired points in the hypoglycemic range (< 90 mg/dl) to complete an analysis (8/136). In the hyperglycemic range (101/136), the algorithm showed an average sensitivity of 91% and an average specificity of 93% against hourly reference. A high sensitivity score indicates that the new hospital CGM has few false alarms for untrue hyper events; and a high specificity score indicates that the new hospital CGM rarely misses a true hyper event.

## Conclusions

Alarms are more than convenient features; they are an important component of product and patient safety. However, poor or inconsistent sensitivity and specificity can quickly diminish the value of an alarm, reducing it to little more than a nuisance. This analysis shows that the novel CGM has the potential to provide sensitivity and specificity to satisfy the demands of the hospital environment. Given the growing reliance on automated and semi-automated clinical systems and the inherent safety implications resulting from this trend, alarm performance should be an important consideration when evaluating these products.

